# Viral aetiology of common colds of outpatient children at primary care level and the use of antibiotics

**DOI:** 10.1590/0074-02760150154

**Published:** 2015-11

**Authors:** Janete Kamikawa, Celso Francisco Hernandes Granato, Nancy Bellei

**Affiliations:** Universidade Federal de São Paulo, Departamento de Medicina, Laboratório de Virologia Clínica, São Paulo, SP, Brasil

**Keywords:** common cold, influenza, respiratory syncytial virus, rhinovirus, antibiotics

## Abstract

Although antibiotics are ineffective against viral respiratory infections, studies have shown high rates of prescriptions worldwide. We conducted a study in Brazil to determine the viral aetiologies of common colds in children and to describe the use of antibiotics for these patients. Children up to 12 years with common colds were enrolled from March 2008-February 2009 at a primary care level facility and followed by regular telephone calls and medical consultations. A nasopharyngeal wash was obtained at enrollment and studied by direct fluorescence assay and polymerase chain reaction for nine different types of virus. A sample of 134 patients was obtained, median age 2.9 years (0.1-11.2 y). Respiratory viruses were detected in 73.9% (99/134) with a coinfection rate of 30.3% (30/99). Rhinovirus was the most frequent virus (53/134; 39.6%), followed by influenza (33/134; 24.6%) and respiratory syncytial virus (8/134; 13.4%). Antibiotic prescription rate was 39.6% (53/134) and 69.8% (37/53) were considered inappropriate. Patients with influenza infection received antibiotics inappropriately in a greater proportion of cases when compared to respiratory syncytial virus and rhinovirus infections (p = 0.016). The rate of inappropriate use of antibiotics was very high and patients with influenza virus infection were prescribed antibiotics inappropriately in a greater proportion of cases.

Common cold syndrome is a very frequent upper respiratory tract infection especially in childhood and can be caused by a variety of different respiratory virus such as rhinovirus, influenza virus, respiratory syncytial virus, adenovirus, metapneumovirus, parainfluenza virus, bocavirus, coronavirus and enterovirus (Ruohola et al. 2009). Despite being regarded as harmless diseases, they can cause significant morbidity and are the most common acute reason for children to visit their primary care physician's office (Ahmed et al. 2010, Schappert & Rechtsteiner 2011). It is well known that antibiotics are ineffective against viral infections and that inappropriate use can put patients at harm for allergic reactions and antibiotic-resistant infections (Grijalva et al. 2009). Although decreasing, recent studies have shown high rates of antibiotic prescription for viral respiratory infections, ranging from 20-60% (El Sayed et al. 2009, Ahmed et al. 2010). Secondary bacterial infections such as sinusitis, acute otitis media (AOM) and even pneumonia can occur after common cold episodes, but most cases of common colds follow a self-limited predictable course and recognising its usual signs and symptoms could avoid unnecessary antibiotic therapy (Dowell et al. 1998b). To combat the spread of resistant bacteria, judicious prescription of antibiotics has become an important target of medical organisations worldwide (Brandileone et al. 2006, Higashi & Fukuhara 2009). In order to describe the viral aetiology of common colds and to best address the problem of antibiotic prescription for these viral respiratory infections in children, we conducted an observational study at an outpatient primary care facility in Brazil.

## PATIENTS, MATERIALS AND METHODS

The study was conducted from 1 March 2008-28 February 2009 at a primary care facility in São Paulo, Southeast Region of Brazil. Children of less than 12 years of age were attended by paediatricians and the clinical diagnosis was coded using the International Classification of Disease, 10th Revision. All children seen on Tuesday's and Friday's mornings with the diagnosis of common cold (J00) or acute upper respiratory infections of multiple and unspecified sites (J06) with symptoms starting in the last five days were included in the study sample. Patients with any associated codes for sinusitis, otitis media, bacterial pharyngitis, pneumonia or who had underlying chronic heart or lung disease or any other chronic health problem or immune disorder were excluded. Patients who had used antibiotics in the last five days were also excluded. The study was approved by the Ethical Committee of São Paulo Federal University (CEP 0670/08) and written informed consent was obtained from parents or guardians before enrollment of each patient.

An independent paediatrician who was not responsible for any clinical intervention filled out a standardised case report form that included information on demographic characteristics and clinical syndrome for each enrolled patient. Data of follow-up were obtained in medical records of subsequent office visits and also by regular telephone calls to the parents every two or three days. All data were entered on the form until the resolution of the respiratory illness. Data about medical prescription of antibiotics, symptoms on the day of antibiotic prescription, laboratory tests and X-ray exams reports were recorded on the form. The use of antibiotics was considered appropriate for all patients with signs and symptoms of secondary bacterial infection diagnosed as AOM, bacterial sinusitis or pneumonia with evidence of parenchymal infiltrates in the chest X-ray exam. For patients with the diagnosis of sinusitis, the use of antibiotics was considered appropriate in cases of symptoms of rhinosinusitis and cough without improvement for more than 10-14 days or more severe upper respiratory tract signs and symptoms, i.e., fever ≥ 39ºC, facial swelling, facial pain (Dowell et al. 1998a).

*Laboratory tests* - A sample of nasopharyngeal wash was obtained from each patient at the enrollment visit. The median time from the onset of symptoms to the collection of nasal washings was three days (1-5 days). Samples were collected and processed using previously published procedures (Carraro et al. 2007) and immediately transported to the laboratory for respiratory virus testing.

Respiratory samples were tested first with the commercial panel SimulFluor Respiratory Screen (Chemicon International, USA) for detection of seven respiratory viruses: respiratory syncytial virus, influenza A and B viruses, parainfluenza virus types 1-3 and adenovirus, as described previously (Bellei et al. 2007, Carraro et al. 2007). Original specimens were then stored at -80°C for further analysis and as polymerase chain reaction (PCR) tests became available in our laboratory, the viral RNA and DNA were extracted from each sample using QIAamp Viral RNA and DNA extraction Kit (Qiagen, USA) according to the manufacturer's instructions. Nucleic acid extracts were tested by PCR for the detection of respiratory syncytial virus, influenza A and B viruses, adenovirus, rhinovirus, metapneumovirus, bocavirus, coronavirus and enterovirus, according to published methods (Allard et al. 2001, Savolainen et al. 2002, Erdman et al. 2003,Falsey et al. 2003, Allander et al. 2005, Brittain-Long et al. 2008, Vijgen et al. 2008, CDC 2009).

*Statistical analysis* - Statistical analysis was performed using SPSS 20.0 and Stata 12 statistical softwares and the statistical significance level for all tests was 5%. The existence of associations between two categorical variables was verified using the chi-square test or the Fisher's exact test depending on the sample size. The Student's *t* test was used to perform comparisons of averages between two groups of data in cases of independent samples. To compare more than two averages, the analysis of variances (ANOVA) was used. Normal distribution of data was previously verified using Kolmogorov-Smirnov test. Nonparametric Mann-Whitney *U* and Kruskal-Wallis tests were used to compare the averages of samples that did not meet the normality assumption. If differences were found between averages, multiple comparisons were performed to localise such differences. For the pairwise comparisons of groups, adjustments in the descriptive levels were made so that the overall significance was 5%.

## RESULTS

During the whole study, 3,282 medical consultations were carried out, 29% (955/3282) of which were coded as common cold or acute upper respiratory infections of multiple and unspecified sites. Of these 955 common cold cases, a sample of 134 patients who met the inclusion criteria was obtained, median age 2.9 years (0.1-11.2 y), 49% male. The most frequent symptoms were coryza (91.8%, 123/134), cough (90.3%, 121/134), fever (56%, 75/134) and wheezing (46.3%, 62/134). Respiratory viruses were detected in 73.9% (99/134) of nasopharyngeal wash samples (1 sample per patient) with a coinfection rate of 30.3% (30/99). The laboratory tests findings are described in Table I.

**TABLE I:** Description of the respiratory virus findings in 134 cases of children with common colds, São Paulo, Brazil, 2008-2009

Virus	Positive samples^*a*^ n (%)
Rhinovirus	30 (22.4)
Respiratory syncytial virus	12 (9)
Influenza A	12 (9)
Influenza B	6 (4.5)
Adenovirus	6 (4.5)
Enterovirus	2 (1.5)
Metapneumovirus	1 (0.7)
Total of monoinfection cases	69 (51.5)
Rhinovirus and influenza A	6 (4.5)
Rhinovirus and adenovirus	4 (3)
Rhinovirus and respiratory syncytial virus	3 (2.2)
Adenovirus and influenza A	3 (2.2)
Rhinovirus and influenza B	2 (1.5)
Rhinovirus and bocavirus	2 (1.5)
Influenza B and coronavirus	2 (1.5)
Rhinovirus and enterovirus	1 (0.7)
Rhinovirus and coronavirus	1 (0.7)
Respiratory syncytial virus and enterovirus	1 (0.7)
Respiratory syncytial virus and coronavirus	1 (0.7)
Total of coinfection cases with 2 viruses	26 (19.4)
Rhinovirus, influenza A and enterovirus	1 (0.7)
Rhinovirus, influenza A and bocavirus	1 (0.7)
Rhinovirus, respiratory syncytial virus and adenovirus	1 (0.7)
Rhinovirus, coronavirus and metapneumovirus	1 (0.7)
Total of coinfection with 3 viruses	4 (3)
Total positive	99 (73.9)
Total negative	35 (26.1)

Total	134 (100)

*a*: numbers of positive samples are a combination of positive direct fluorescence, polymerase chain reaction (PCR) and reverse transcription-PCR results.

Overall, the antibiotic prescription rate was 39.6% (53/134), among which 60.4% (32/53) was amoxicillin, 22.6% (12/53) macrolides, 9.4% (5/53) cephalosporins and 7.6% (4/53) amoxicillin plus sulbactam (Table II). Of 53 patients who received antibiotics during the follow-up, only 30.2% (16/53) received them judiciously and the other 69.8% (37/53) received them inappropriately. Among these 37 cases with inappropriate use, the clinical justifications for prescription of antibiotics were: in 37.7% (20/53) to treat nasal or postnasal discharge during the first week of common cold symptoms in patients without fever, in 18.9% (10/53) to treat persistence of cough during the first week of symptoms, in 11.3% (6/53) to treat common cold and in 1.9% (1/53) to treat wheezing symptom.

**TABLE II:** Laboratory findings and prescription of antibiotics during the follow-up of children with common colds

	Age (years)	Virus	Antibiotic	Day of antibiotic introduction^*a*^
Judicious prescription				
Sinusitis	0.5	RV	AZI	9
	1.7	RV	AMO	14
	1.8	RV	AMO	18
	3	RV/IFA/AdV	AMO	10
	3	NEG	AMO	19
	5.6	RV	AMO	4
	7.1	RV/IFB	CCL	5
Acute otitis media	0.4	RSV	AMO	12
	0.5	RV/IFA	AMO	4
	0.6	RV/BoV	AMO	5
	0.7	NEG	AMO	3
	1.1	RSV	AMO	5
	4.8	NEG	AZI	8
Pneumonia	0.6	NEG	AMO	10
	0.7	RV	CLAR	12
	0.9	NEG	AMO + SB	4
Inappropriate prescription				
Common cold	6.2	RV	AMO	1
	0.7	IFB	AMO	1
	2.2	IFA	AMO	2
	0.6	IFB	ERY	3
	2.1	IFA	AMO + SB	3
Nasal /postnasal secretion	7.8	NEG	AMO	2
	4.3	IFB	CEX	3
	5.3	RV	AMO	3
	2	RSV	AMO	3
	2	RSV	AMO	3
	5.5	NEG	AZI	3
	3.9	RV/IFA	AZI	3
	8.7	IFA/AdV	CEX	4
	0.5	RSV/RV	AMO	4
	4.1	RV/IFA	AZI	4
	6.6	IFA	AMO	4
	3.2	RV/BoV/IFA	AMO	4
	8.1	IFB	AMO	5
	6.7	RV/Cov	AMO	5
	6.7	RV/IFA	AMO	5
	8.8	IFA	AZI	5
	6.5	RSV	AMO	5
	11.2	NEG	AMO	6
Cough	1.1	RV/EV/IFA	AMO	2
	7.2	NEG	AZI	2
	9.7	NEG	CEX	2
	0.4	NEG	CLAR	2
	0.5	RSV/AdV/RV	AMO	3
	7.3	NEG	CEX	3
	0.7	NEG	AMO	3
	0.5	RV	AMO	3
	3.8	NEG	AMO + SB	4
	3.8	IFA	AMO + SB	5
	1.1	IFA	AMO	6
	0.2	RSV/RV	AMO	7
Wheezing	0.8	RV	AMO	2
Pharyngitis	2.9	IFB/Cov	AMO	6

*a*: day 1 was the first day of common cold symptoms; AdV: adenovirus; AMO: amoxicillin; AZI: azithromycin; BoV: bocavirus; CCL: cefaclor; CEX: cephalexin; CLAR: clarithromycin; Cov: coronavirus; ERY: erythromycin; EV: enterovirus; IFA: influenza A; IFB: influenza B; NEG: negative; RSV: respiratory syncytial virus; RV: rhinovirus; SB: sulbactam.

Among 75 children who had fever at the onset of symptoms, 45.3% (34/75) were prescribed antibiotics whereas 32.2% (19/59) of those who did not have fever at the onset of symptoms received antibiotics. Thus, there was no difference in the proportion of antibiotic prescriptions between children who had fever at the onset of symptoms and those who did not (p = 0.123).

Of a total of 53 children who received antibiotics, 34 presented fever at onset of symptoms and of these, 29.4% (10/34) received judicious prescription of antibiotics. Of the remaining 19 children who did not have fever at the onset of common cold symptoms and received antibiotics, 31.6% (6/19) were prescribed antibiotics judiciously. Thus, there was also no difference for judicious prescription of antibiotics between children with fever and those without fever at the onset of common cold symptoms (p = 0.869).

The average time to the resolution of symptoms of children with signs of secondary bacterial infection was of 16.7 days and, within this group, all children received antibiotics. Among children with no signs of bacterial infection, the average time to the resolution of symptoms was 8.9 days for the group that received antibiotics and 7.0 days for the group that did not.

Among patients with respiratory virus monoinfection, all patients with influenza received antibiotics inappropriately (10/10), whereas those with respiratory syncytial virus were prescribed antibiotics inappropriately in 60% (3/5) and those with rhinovirus were prescribed antibiotics inappropriately in 44.4% of cases (5/9) (p = 0.016). Also, of seven patients coinfected with influenza, 71.4% (5/7) received antibiotics inappropriately, as showed in Table II. None of the patients were vaccinated against influenza by the time of the study.

## DISCUSSION

Inappropriate use of antibiotics for viral common colds is an important problem worldwide but, until now, we did not have clinical studies addressing this problem in Brazil. During the clinical course of a common cold, secondary bacterial infections may occur, however, antimicrobials are frequently used inappropriately for events that are normal during the clinical course of a viral infection (Dowell et al. 1998b). In our study, this practice was particularly evident in cases of viral rhinosinusitis, since 37.7% (20/53) of patients received antibiotics for the presence of nasal or postnasal discharge during the first week of common cold symptoms without fever. During childhood, 20-40% of common colds can be complicated by AOM (Wald et al. 1991, Chonmaitree et al. 2008) and although current recommendations on management of AOM encourage the initial observation of nonsevere AOM cases in selected children (i.e., “watchful waiting”) (AAP 2004), there are still controversies around the diagnosis and management of AOM (Hoberman et al. 2011,Shaikh et al. 2011). In routine practice, the over diagnosis of AOM is frequent but, considering that there are difficulties in confirming the diagnosis, in our study we considered all indications of antibiotic for AOM as adequate regardless of the patients' age, severity of symptoms and certainty of diagnosis. We believe that rates of inappropriate use of antibiotics could be even greater if stringent diagnostic criteria were applied for AOM diagnosis.

Amoxicillin was the first choice for antibiotic therapy accounting for 60.4% (32/53) of all prescriptions but, on the other hand, the second most prescribed antibiotics were macrolides (22.6%, 12/53) which is not in accordance with current guidelines (Bradley et al. 2011). Predominantly in the paediatric outpatient population, the growing emergence of bacterial resistance to macrolides is related to delayed cure and is responsible for a great proportion of costs currently associated with antibiotic resistant pneumococcal infections (Kawai et al. 2012, Reynolds et al. 2014). Continuing medical education programs aimed not only to reduce the inappropriate use of antibiotics, but also to teach the best practices of antibiotic therapy could be of great help to improve this scenario.

Diagnostic uncertainty, which is defined as the difficulty in distinguishing a self-limited viral infection from a bacterial infection requiring antibiotic therapy is identified by researchers as a factor that contributes for antibiotic overuse (Arnold et al. 2005). Thus, the difficulty in predicting a bacterial infection in febrile children could lead to the misuse of antibiotics. Bacterial infections are associated with worsening of clinical conditions with a consequent longer time for the resolution of symptoms. However, common colds without secondary infections follow a self-limited course of seven-10 days with no reduction of the duration of symptoms with the use of antibiotic, as it was observed in this study and as reported elsewhere in the medical literature (Kaiser et al. 2001, Brandileone et al. 2006, Li et al. 2009, Carranza-Martinez et al. 2010).

Although our study was not designed to evaluate the influence of fever in medical decision to prescribe antibiotics, we observed that there was no difference in the proportion of antibiotic prescriptions for patients who had fever at the onset of symptoms and for those who did not, as well as in the proportion of judicious prescription of antibiotics for these two groups of patients. Additional research is needed to identify the local factors that are determinants of antibiotic misuse.

Although it is well known that influenza vaccination is the primary strategy to prevent influenza, the Brazilian Health Ministry started public vaccination campaigns against influenza for children in 2010 so that part of the population had no access to influenza vaccine by the time of the study. None of the patients of the study were vaccinated against influenza and this fact could explain the high frequency of influenza infection in our patients (23.1%; 31/134), as showed in Table I.

Antiviral treatment also plays an important role in decreasing influenza-related morbidity and mortality, but none of 33 patients with laboratory confirmed influenza infection received antiviral therapy, whereas all patients with influenza monoinfection received antibiotics inappropriately (10/10) and those coinfected with influenza and other viruses also received antibiotics inappropriately in a great proportion of cases (71.4%; 5/7). A recent study in the United States of America reported that during 2012-2013, antiviral medications were underprescribed and antibiotics may have been inappropriately prescribed to a large proportion of outpatients with influenza (Havers et al. 2014). The referred study emphasises that the use of sensitive and specific tests for the rapid diagnosis of influenza and other respiratory viruses is strongly recommended and could decrease antibiotic use and guide appropriate use of antiviral agents in both outpatient and inpatient settings (Bradley et al. 2011). In Brazil, diagnostic tests for respiratory virus are still expensive and are not easily available for the vast majority of the population, especially for low income people.

This study was conducted at one primary care facility and it is not possible to generalise the results to all Brazilian primary health care services, however, these preliminary data will help us to better understand antibiotic misuse among children with common cold viral infections. We believe that continuing education on appropriate antibiotics and antivirals use as well as accessibility to influenza vaccination, sensitive and specific tests for the rapid diagnosis of respiratory viruses and antiviral medication are essential to improve primary healthcare quality.

## References

[B1] AAP - American Academy of Pediatrics (2004). Diagnosis and management of acute otitis
media. Pediatrics.

[B2] Ahmed MN, Muyot MM, Begum S, Smith P, Little C, Windemuller FJ (2010). Antibiotic prescription pattern for viral respiratory
illness in emergency room and ambulatory care settings. Clin Pediatr (Phila).

[B3] Allander T, Tammi MT, Eriksson M, Bjerkner A, Tiveljung-Lindell A, Andersson B (2005). Cloning of a human parvovirus by molecular screening of
respiratory tract samples. Proc Natl Acad Sci USA.

[B4] Allard A, Albinsson B, Wadell G (2001). Rapid typing of human adenoviruses by a general PCR
combined with restriction endonuclease analysis. J Clin Microbiol.

[B5] Arnold SR, To T, McIsaac WJ, Wang EEL (2005). Antibiotic prescribing for upper respiratory tract
infection: the importance of diagnostic uncertainty. J Pediatr.

[B6] Bellei N, Carraro E, Perosa AHS, Benfica D, Granato CFH (2007). Influenza and rhinovirus infections among health-care
workers. Respirology.

[B7] Bradley JS, Byington CL, Shah SS, Alverson B, Carter ER, Harrison C, Kaplan SL, Mace SE, McCracken GH, Moore MR, St Peter SD, Stockwell JA, Swanson JT (2011). Executive summary: the management of community-acquired
pneumonia in infants and children older than 3 months of age: clinical practice
guidelines by the Pediatric Infectious Diseases Society and the Infectious
Diseases Society of America. Clin Infect Dis.

[B8] Brandileone MCC, Casagrande ST, Guerra MLLS, Zanella RC, Andrade ALSS, Fabio JLD (2006). Increase in numbers of beta-lactam-resistant invasive
Streptococcus pneumoniae in Brazil and the impact of conjugate vaccine
coverage. J Med Microbiol.

[B9] Brittain-Long R, Westin J, Olofsson S, Lindh M, Andersson LM (2008). Multiplex real-time PCR for detection of respiratory
tract infections. J Clin Virol.

[B10] Carranza-Martinez MI, Newton-Sanchez O, Franco-Paredes C, Villaseñor-Sierra A (2010). Clinical outcomes in Mexican children with febrile acute
upper respiratory tract infections: no impact of antibiotic
therapy. Int J Infect Dis.

[B11] Carraro E, Ferreira D, Benfica D, Perosa AHS, Granato CFH, Bellei NCJ (2007). Applications of a duplex reverse transcription
polymerase chain reaction and direct immunofluorescence assay in comparison with
virus isolation for detection of influenza A and B. Diagn Microbiol Infect Dis.

[B12] CDC - Centers for Disease Control and Prevention (2009). CDC protocol of realtime RT-PCR for influenza A (H1N1). CDC
realtime RT-PCR (rRT-PCR) protocol for detection and characterization of swine
influenza (version 2009).

[B13] Chonmaitree T, Revai K, Grady JJ, Clos A, Patel JA, Nair S, Fan J, Henrickson KJ (2008). Viral upper respiratory tract infection and otitis media
complication in young children. Clin Infect Dis.

[B14] Dowell SF, Schwartz B, Phillips WR (1998a). Appropriate use of antibiotics for URIs in children.
Part I. Otitis media and acute sinusitis. The Pediatric URI Consensus
Team. Am Fam Physician.

[B15] Dowell SF, Schwartz B, Phillips WR (1998b). Appropriate use of antibiotics for URIs in children.
Part II. Cough, pharyngitis and the common cold. The Pediatric URI Consensus
Team. Am Fam Physician.

[B16] El Sayed MF, Tamim H, Jamal D, Mumtaz G, Melki I, Yunis K, National Collaborative Perinatal Neonatal Network (NCPNN) (2009). Prospective study on antibiotics misuse among infants
with upper respiratory infections. Eur J Pediatr.

[B17] Erdman DD, Wenberg GA, Edwards KM, Walker FJ, Anderson BC, Winter J, González M, Anderson LJ (2003). GeneScan reverse transcription-PCR assay for detection
of six common respiratory viruses in young children hospitalized with acute
respiratory illness. J Clin Microbiol.

[B18] Falsey AR, Erdman D, Anderson LJ, Walsh EE (2003). Human metapneumovirus infections in young and elderly
adults. J Infect Dis.

[B19] Grijalva CG, Nuorti JP, Griffin MR (2009). Antibiotic prescription rates for acute respiratory
tract infections in US ambulatory settings. JAMA.

[B20] Havers F, Thaker S, Clippard JR, Jackson M, McLean HQ, Gaglani M, Monto AS, Zimmerman RK, Jackson L, Petrie JG, Nowalk MP, Moehling KK, Flannery B, Thompson MG, Fry AM (2014). Use of influenza antiviral agents by ambulatory care
clinicians during the 2012-2013 influenza season. Clin Infect Dis.

[B21] Higashi T, Fukuhara S (2009). Antibiotic prescriptions for upper respiratory tract
infection in Japan. Intern Med.

[B22] Hoberman A, Paradise JL, Rockette HE, Shaikh N, Wald ER, Kearney DH, Colborn DK, Kurs-Lasky M, Bhatnagar S, Haralam MA, Zoffel LM, Jenkins C, Pope MA, Balentine TL, Barbadora KA (2011). Treatment of acute otitis media in children under 2
years of age. N Engl J Med.

[B23] Kaiser L, Morabia A, Stalder H, Ricchetti A, Auckenthaler R, Terrier F, Hirschel B, Khaw N, Lacroix JS, Lew D (2001). Role of nasopharyngeal culture in antibiotic
prescription for patients with common cold or acute sinusitis. Eur J Clin Microbiol Infect Dis.

[B24] Kawai Y, Miyashita N, Yamaguchi T, Saitoh A, Kondoh E, Fujimoto H, Teranishi H, Inoue M, Wakabayashi T, Akaike H, Ogita S, Kawasaki K, Terada K, Kishi F, Ouchi K (2012). Clinical efficacy of macrolide antibiotics against
genetically determined macrolide-resistant Mycoplasma pneumoniae pneumonia in
paediatric patients. Respirology.

[B25] Li J, De A, Ketchum K, Fagnan LJ, Haxby DG, Thomas A (2009). Antimicrobial prescribing for upper respiratory
infections and its effect on return visits. Fam Med.

[B26] Reynolds CA, Finkelstein JA, Ray GT, Moore MR, Huang SS (2014). Attributable healthcare utilization and cost of
pneumonia due to drug-resistant streptococcus pneumonia: a cost
analysis. Antimicrob Resist Infect Control.

[B27] Ruohola A, Waris M, Allander T, Ziegler T, Heikkinen T, Ruuskanen O (2009). Viral etiology of common cold in children,
Finland. Emerg Infect Dis.

[B28] Savolainen C, Mulders MN, Hovi T (2002). Phylogenetic analysis of rhinovirus isolates collected
during successive epidemic seasons. Virus Res.

[B29] Schappert SM, Rechtsteiner EA (2011). Ambulatory medical care utilization estimates for
2007. Vital Health Stat.

[B30] Shaikh N, Hoberman A, Kaleida PH, Rockette HE, Kurs-Lasky M, Hoover H, Pichichero ME, Roddey OF, Harrison C, Hadley JA, Schwartz RH (2011). Otoscopic signs of otitis media. Pediatr Infect Dis J.

[B31] Vijgen L, Moës E, Keyaerts E, Li S, Van Ranst M (2008). A pancoronavirus RT-PCR assay for detection of all known
coronaviruses. Methods Mol Biol.

[B32] Wald ER, Guerra N, Byers C (1991). Upper respiratory tract infections in young children:
duration of and frequency of complications. Pediatrics.

